# Structure of the Non-Catalytic Domain of the Protein Disulfide Isomerase-Related Protein (PDIR) Reveals Function in Protein Binding

**DOI:** 10.1371/journal.pone.0062021

**Published:** 2013-04-16

**Authors:** Roohi Vinaik, Guennadi Kozlov, Kalle Gehring

**Affiliations:** Department of Biochemistry, Groupe de Recherche Axé sur la Structure des Protéines, McGill University, Montréal, Québec, Canada; University of Oulu, Finland

## Abstract

Protein disulfide isomerases comprise a large family of enzymes responsible for catalyzing the proper oxidation and folding of newly synthesized proteins in the endoplasmic reticulum (ER). Protein disulfide isomerase-related (PDIR) protein (also known as PDIA5) is a specialized member that participates in the folding of α_1_-antitrypsin and N-linked glycoproteins. Here, the crystal structure of the non-catalytic domain of PDIR was determined to 1.5 Å resolution. The structure adopts a thioredoxin-like fold stabilized by a structural disulfide bridge with a positively charged binding surface for interactions with the ER chaperones, calreticulin and ERp72. Crystal contacts between molecules potentially mimic the interactions of PDIR with misfolded substrate proteins. The results suggest that the non-catalytic domain of PDIR plays a key role in the recognition of protein partners and substrates.

## Introduction

In order to acquire the proper arrangement of disulfide bonds, newly synthesized membrane and secreted proteins fold in the oxidative environment of the endoplasmic reticulum (ER) with the assistance of ER enzymes called protein disulfide isomerases (PDIs) [1], [2], [3]. The PDI proteins are defined by their ER localization and the presence of one or more thioredoxin-like domains, which usually possess oxidoreductase activity mediated by a conserved catalytic CxxC motif. Within the catalytic motif, the N-terminal cysteine forms a mixed disulfide with protein substrate, and the C-terminal cysteine mediates release of the substrate [4]. Most PDI family members contain additional non-catalytic thioredoxin-like domains that lack the CxxC motif. Some of these domains are involved in substrate or co-chaperone recognition, while others may simply function as spacers for positioning catalytic elements (for a review, see [3]).

Human protein disulfide isomerase-related (PDIR) protein, also known as PDIA5, was originally identified from a human placental cDNA library [5]. Previous studies have demonstrated that PDIR is up-regulated when mouse liver cells are exposed to tunicamycin, a drug that inhibits *N*-linked glycosylation of nascent polypeptides during protein synthesis in the ER [5]. PDIR is also up-regulated in mucopolysaccharidoses, lysosomal storage diseases caused by defects in lysosomal enzymes involved in degrading glycosaminoglycans [6]. Screening for human PDIR-binding proteins using a T7 phage display system revealed that α_1_-antitrypsin binds to PDIR [7]. A second hit was the lectin-like chaperone calreticulin; surface plasmon resonance experiments showed moderate binding affinity between calreticulin and PDIR. This was corroborated by a recent mass-spectrometry-based study, which additionally identified ERp72 as a PDIR-interacting protein [8]. Catalytically, PDIR demonstrates lower isomerase and chaperone activity than the archetypal PDI (PDIA1) but is more efficient in the oxidative refolding of α_1_-antitrypsin [7].

PDIR is the only PDI composed of an N-terminal non-catalytic domain and three catalytic domains. The catalytic domains are unique in the sequences of their catalytic motifs: CSMC, CGHC and CPHC. Of these domains, the second catalytic domain (CGHC) exhibited the highest isomerase activity on model substrates, while the CSMC domain was most important for the folding of α_1_-antitrypsin [7]. Interestingly, replacement of the non-catalytic domain of PDIR with the substrate-binding **b**'domain of PDIA1 increases the chaperone activity of PDIR to PDIA1 levels, but decreases oxidative refolding of α_1_-antitrypsin [9]. This suggests that in a fashion analogous to other PDI proteins, the non-catalytic domain of PDIR plays a key role in binding substrates and chaperone partners. Despite its unique functional properties, there are no structures of the domains of PDIR, and the molecular basis for substrate and partner recognition is unknown.

Here, we present the crystal structure of the non-catalytic domain of human PDIR, determined at 1.5 Å resolution. The structure reveals a highly conserved positively charged surface that is involved in interactions with protein partners. NMR titrations show that the domain binds to the negatively charged P-domain of calreticulin, which suggests that PDIR can engage the calnexin cycle. Crystal contacts between PDIR molecules further identify a putative substrate-binding site on the PDIR non-catalytic domain. The observed interactions suggest that PDIR can bind substrates directly and also recruit them through interaction with calreticulin.

## Materials and Methods

### Protein expression, preparation and purification

The non-catalytic domain (residue 29–150) of human PDIR was cloned into pET29a (Amersham-Pharmacia) and expressed in *E. coli* BL21(DE3) in rich (LB) medium as a C-terminal His-tag fusion. For production of selenomethionine-labeled protein, the expression plasmid was transformed into the *E. coli* methionine auxotroph strain DL41 (DE3) and the protein was produced using LeMaster medium [10]. Cells were harvested and broken in 50 mM HEPES, 500 mM NaCl, 5% (v/v) glycerol, pH 7.5. The protein was purified by affinity chromatography on Ni^2+^-charged Sepharose resin followed by size-exclusion chromatography using HPLC buffer (10 mM HEPES, 100 mM NaCl, 5 mM dithiothreitol, pH 7.0). Selenomethionine-labeled protein was purified in a similar manner. In addition to the PDIR sequence, the resulting protein contains an N-terminal methionine and a C-terminal extension Leu-Glu-His-His-His-His-His-His. The ^15^N-labeled P-domain of mouse calreticulin (residues 211–261) was expressed and purified as described earlier [11].

### Crystallization

Initial crystallization conditions for the PDIR non-catalytic domain were identified utilizing hanging drop vapor diffusion using PACT screen (QIAGEN). The best crystals were obtained by equilibrating a 1.0 µL drop of a protein (6 mg/mL) in buffer (10 mM HEPES (pH 7.0), 0.1 M NaCl and 5 mM DTT), mixed with 1.0 µL of reservoir solution containing 0.15 M DL-malic acid (pH 7.0) and 20% (w/v) PEG 3350, and suspended over 1 mL of reservoir solution. Crystals grew in 3–14 days at 22°C. The identical conditions were used for growing selenomethionine-labeled protein. For cryoprotection, the reservoir composition with the addition of 20% glycerol was used. For data collection, crystals were picked up in a nylon loop and flash cooled in a N_2_ cold stream (Oxford Cryosystem). The crystals belong to the p2_1_2_1_2_1_ space group and contain one molecule in the asymmetric unit (Z  =  4) corresponding to V_m_  =  2.1 Å^3^ Da^−1^ and a solvent content of 42%.

### Structure solution and refinement

Diffraction data from a SeMet-labeled crystal were collected using a single-wavelength (0.9791 Å) SAD regime on a MarMosaic CCD 300 detector at beamline 08ID-1 at the Canadian Light Source, SK, Canada. Diffraction data from a native crystal were collected at a wavelength of 0.9770 Å on an ADSC Quantum-210 CCD detector (Area Detector Systems Corp.) at beamline A1 at the Cornell High-Energy Synchrotron Source (CHESS) (Table 1). Data processing and scaling were performed with HKL2000 [12]. The structure was determined by SAD phasing using the program PHENIX [13] resulting in automated model building of approximately 90% of the residues. The partial model obtained from PHENIX was extended manually with the help of the program Coot [14] and was improved by several cycles of refinement using the program REFMAC [15] and model refitting. The obtained structure was used to phase the native diffraction dataset. The final model has good stereochemistry with no outliers in the Ramachandran plot computed using PROCHECK [16]. Figures were made with PyMOL (http://pymol.sourceforge.net/). The coordinates and structure factors have been deposited in the RCSB Protein Data Bank (accession number 4i6x).

**Table 1:** Data collection and refinement statistics.

Data collection	SeMet dataset	native dataset
Space group	P2_1_2_1_2_1_	P2_1_2_1_2_1_
Cell dimensions		
*a*, *b*, *c* (Å)	34.7, 41.1, 88.7	34.69, 41.27, 90.89
Wavelength (Å)	0.9795	0.9770
Resolution (Å)	50–1.63 (1.67–1.63)^1^	50–1.50 (1.53–1.50)
*R*_sym_	0.060 (0.854)	0.031 (0.268)
*<I* / σ*I>*	15.6 (1.3)	43.5 (3.6)
Completeness (%)	78.6 (31.6)	95.3 (67.2)
Redundancy	6.5 (3.5)	5.7 (4.1)
FOM	0.59	
Refinement		
Resolution (Å)		45.5–1.50
No. reflections		19332
*R*_work_ / *R*_free_		0.191/0.237
No. atoms		
Protein		945
Water		125
*B*-factors (Å^2^)		
Protein		17.1
Water		36.3
R.m.s deviations		
Bond lengths (Å)		0.008
Bond angles (°)		1.17
E.S.U. (Å)^2^		0.045
Ramachandran statistics (%)^3^		
Most favored regions		95.5
Additional allowed regions		4.5

1 Highest resolution shell is shown in parentheses.

2 E.S.U.—estimated overall coordinate error based on maximum likelihood.

3 Stereochemistry was computed using PROCHECK.

### NMR spectroscopy

NMR samples contained 0.15–0.5 mM protein in 10 mM HEPES, 100 mM NaCl at pH 7.0. For NMR titrations, unlabeled non-catalytic domain of PDIR was added to ^15^N-labeled 0.15 mM P-domain of calreticulin to the final molar ratio of 1∶4.4, or the unlabeled P-domain was added to ^15^N-labeled 0.15 mM non-catalytic domain of PDIR to the final molar ratio of 1∶8. For the high-salt titration, the proteins were exchanged into 10 mM HEPES, 0.5 M ammonium sulfate at pH 7.0 and the ^15^N-labeled P-domain was titrated with unlabeled PDIR domain to the final molar ratio of 1∶2. All NMR experiments were performed at 298 K using Varian 500 and 800 MHz spectrometers. NMR spectra were processed using NMRPipe [17] and analyzed with SPARKY [18]. NMR resonance assignments of the P-domain of calreticulin were adopted from Biological Magnetic Resonance Bank entry 5204.

## Results

### Occurrence of the non-catalytic PDIR domain in other proteins

**Figure 1:**
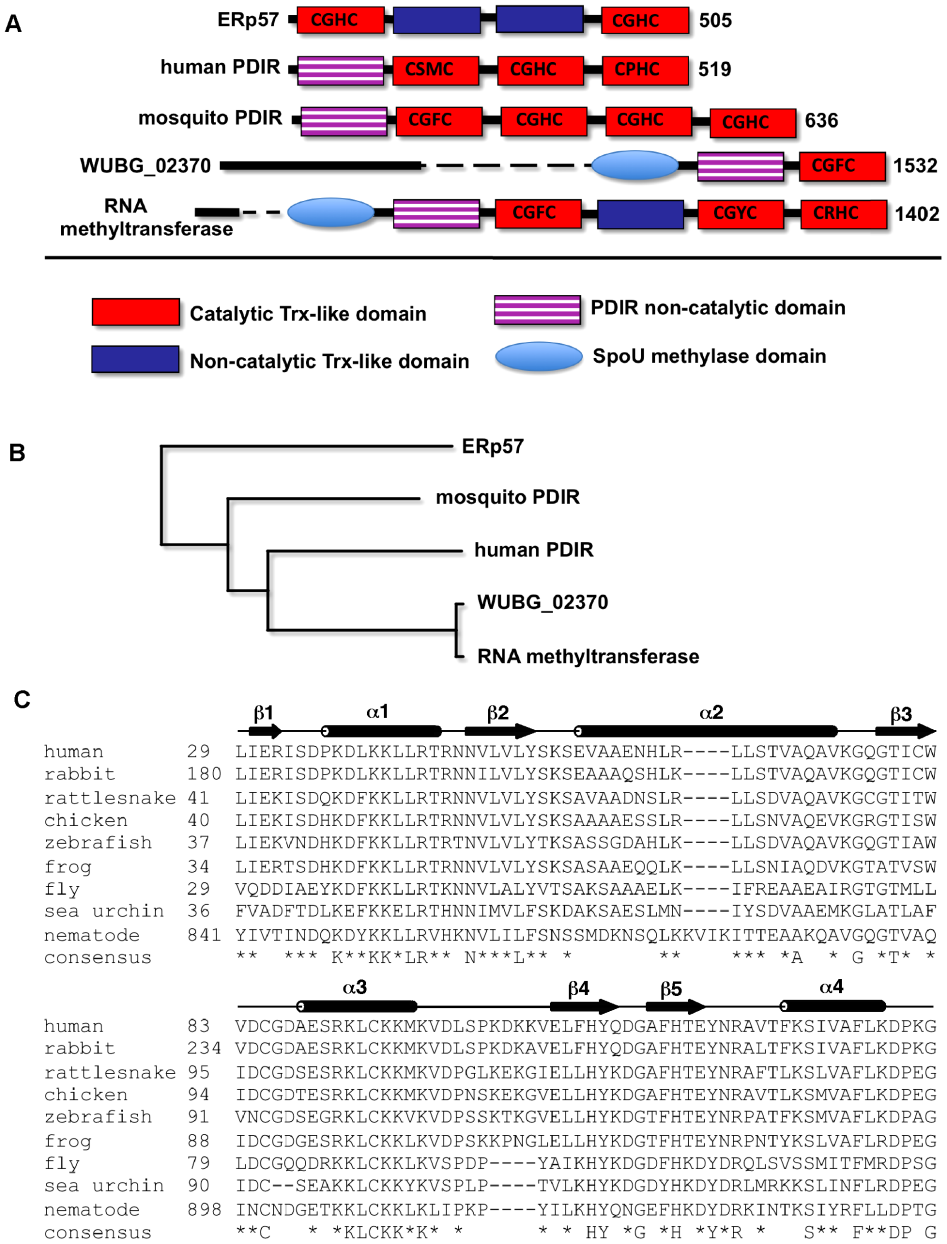
Sequence analysis of the PDIR non-catalytic domain. (A) Occurrence of the domain in protein disulfide isomerases and other proteins. Human ERp57 is shown for comparison. Catalytic motifs are shown in catalytically-active thioredoxin-like domains. (B) Rooted phylogenetic tree of proteins shown in panel (A). Sequences labeled WUBG_02370 and RNA methyltransferase are proteins from parasitic nematodes *Wuchereria bancrofti* (EJW86719) and *Brugia malayi* (XP_001896925); mosquito PDIR is from *Aedes aegypti* (XP_001659136). The N-terminal catalytic domain of ERp57 was used for the phylogenetic tree. The figure was generated with ClustalW [29] and TreeViewPPC [30]. (C) Sequence alignment of the non-catalytic domain from PDIR proteins from human (NP_006801), rabbit (XP_002716857), rattlesnake (AFJ50881), chicken (XP_422097), zebrafish (XP_001107048), frog (XP_001086600), fly (XP_609645), and sea urchin (XP_001200801) and the related sequence from *Brugia malayi* RNA methyltransferase (XP_001896925). The consensus sequence is shown below; the secondary structure elements are above the sequence.

The domain organization of PDIR is unusual. Unlike the better known PDI family members, which contain four thioredoxin-like domains, the N-terminal domain of PDIR is non-catalytic. We used a BLAST sequence similarity search to identify homologous domains. As expected, most of the hits corresponded to PDIR orthologs from different species (Fig. 1). Some of the orthologs contained additional C-terminal catalytic domains; the mosquito protein contains four catalytic domains and the ortholog from the fresh water polyp *Hydra magnipapillata* (XP_002159276) possesses eight. Within the family of PDI proteins, the PDIR non-catalytic domain is relatively unique with a sequence similarity of only 20% identity to its closest paralog, the **a** domain of ERp57.

Interestingly, the search also identified an association of the non-catalytic PDIR domain with SpoU rRNA methylase domains in several roundworms. The methylase domain mediates the methylation of RNA bases and is found immediately adjacent to PDIR-like sequences in *Wuchereria bancrofti* and *Brugia malayi*. Mammalian RNA methyltransferases do not contain PDIR-like domains. Human PDIR has 43% sequence identity to the corresponding domains in the methyltransferases with a preponderance of conserved positively charged residues (Fig. 1).

### Crystallization and structure determination

In order to obtain insight into the structure and function of the non-catalytic domain of PDIR, we screened crystallization conditions using the full-length mature protein and different truncated versions. We were able to obtain crystals of the N-terminal non-catalytic domain (residues 29–150) with crystals appearing in 1–2 days. Unfortunately, the crystals had visible defects and yielded an anisotropic diffraction pattern. The use of a vector with a C-terminal His-tag improved the protein expression and the quality of the crystals.

The low sequence similarity of PDIR to known structures prevented us from using molecular replacement for phasing of the diffraction data. We produced selenomethionine-labeled crystals and determined the structure using a single-wavelength anomalous dispersion (SAD). Consequently, this model was used to refine the native protein structure at 1.50 Å resolution (Table 1). The asymmetric unit contains one protein molecule. The final model did not include the C-terminal residues Glu145-Ala150 and the 8-residue His-tag, which were disordered.

### Structure of the non-catalytic PDIR domain

**Figure 2:**
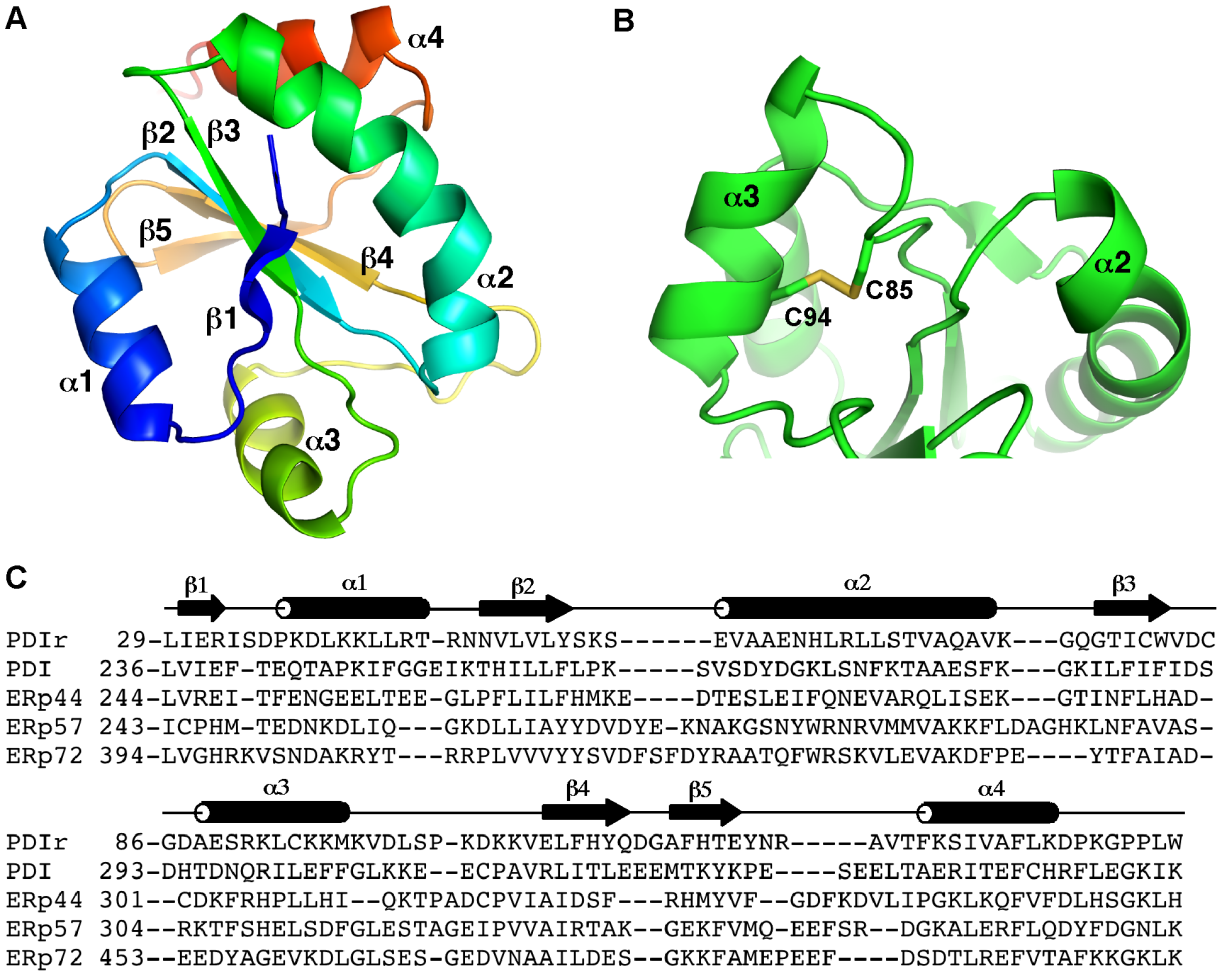
Structure of the PDIR non-catalytic domain. (A) Cartoon representation of the structure, color-coded from the N-terminus (blue) to C-terminus (red). (B) The structural disulfide bridge between Cys85 and Cys94 connects helix α3 with the preceding loop. (C) Structure-based alignment with the non-catalytic domains of other PDIs with known structure. The secondary structure of the PDIR domain is shown.

The structure shows a typical thioredoxin-like fold containing a five-stranded central β-sheet in the β1˜ββ3˜ββ2˜ββ4˜ββ5 arrangement; all the strands are parallel except for strand β4. The β-sheet is flanked on both sides by two α-helices that are parallel to each other (Fig. 2A). The domain boundaries extend from Leu29 to Lys139, which hydrogen bonds with carbonyl of Phe134 through its side chain. The domain does not possess the catalytic CxxC motif but contains three cysteine residues. Two of them, Cys85 and Cys94, form a disulfide bond that connects helix α3 with the preceding loop (Fig. 2B). The third cysteine Cys81 is located at the base of the groove between strand β1 and helix α1. The side chain of this residue has two alternative conformations, one directed into a hydrophobic core and another that is solvent-exposed. Cys81 is unlikely to form functionally relevant disulfides with other proteins as it is poorly conserved in other PDIR proteins (Fig. 1C). The structure allowed us to make a structure-based sequence alignment with the non-catalytic domains of other PDI family members (Fig. 2C). The notoriously low sequence identity between these domains makes reliable sequence-based alignment very difficult.

Structural similarity search DALI [19] identified numerous hits containing protein disulfide isomerases and single-domain thioredoxin proteins. The highest structural similarity was to the N-terminal, catalytic domain **a** of human ERp57 (PDB entry 3f8u; Z-score of 14.7)[20] and the corresponding domain of yeast PDI (PDB entry 2b5e; Z-score of 14.2)[21]. These hits were followed by several thioredoxins and the third catalytic domain of ERp46 (PDB entry 3uvt; Z-score of 14.0)[22].

**Figure 3:**
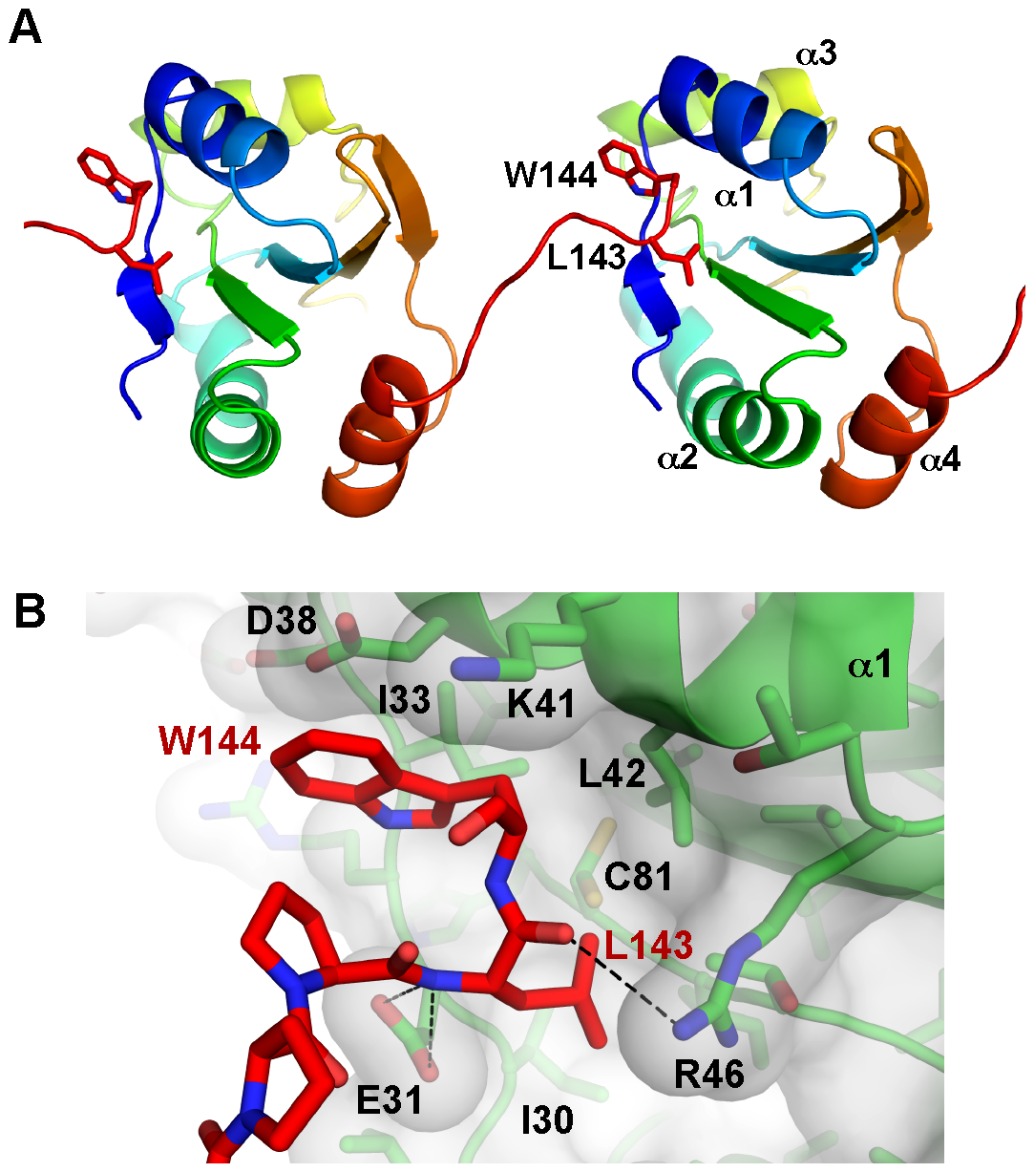
Crystal contacts identify a putative binding surface for hydrophobic polypeptides. (A) The Leu143 and Trp144 from the C-terminal tail of the crystallized fragment bind to a pocket in an adjacent PDIR molecule. (B) The base of the pocket is lined with hydrophobic residues, while Glu31 and Arg46 make hydrogen bonds with backbone amide and carbonyl groups.

We analyzed intermolecular contacts in the crystal for physiologically relevant protein-protein binding surfaces. The C-terminal tail of the PDIR construct is instrumental in crystal formation wherein the side chains of Leu143 and Trp144 insert into a pocket between strand β1 and helix α1 of an adjacent PDIR domain (Fig. 3A). The walls of the pocket are formed by the aliphatic parts of Glu31, Asp38, Lys41 and Arg46 while the hydrophobic side chains of Ile30, Ile33, Leu42 and Cys81 occupy the base of the cavity (Fig. 3B). These hydrophobic contacts are reminiscent of the **b'x** structure of human PDIA1, where a tryptophan residue of the **x** linker between the **b'** and **a'** domains inserts into the substrate-binding site on the non-catalytic **b'** domain [23]. A similar interaction was observed in the recent structure of the third catalytic domain of ERp46 [22]. Importantly, the site of these interactions is different, as the substrate-binding pocket is located between helices α1 and α3 of PDIA1. NMR titrations did not show binding between the PDIR non-catalytic domain and mastoparan, a model hydrophobic peptide that interacts with PDIA1 (data not shown). This suggests that the PDIR substrate-binding pocket has unique specificity, which is distinct from PDIA1 [24]. We suggest that the intermolecular interactions observed in our crystal mimic substrate binding.

### Structure reveals highly conserved positively charged surface

**Figure 4:**
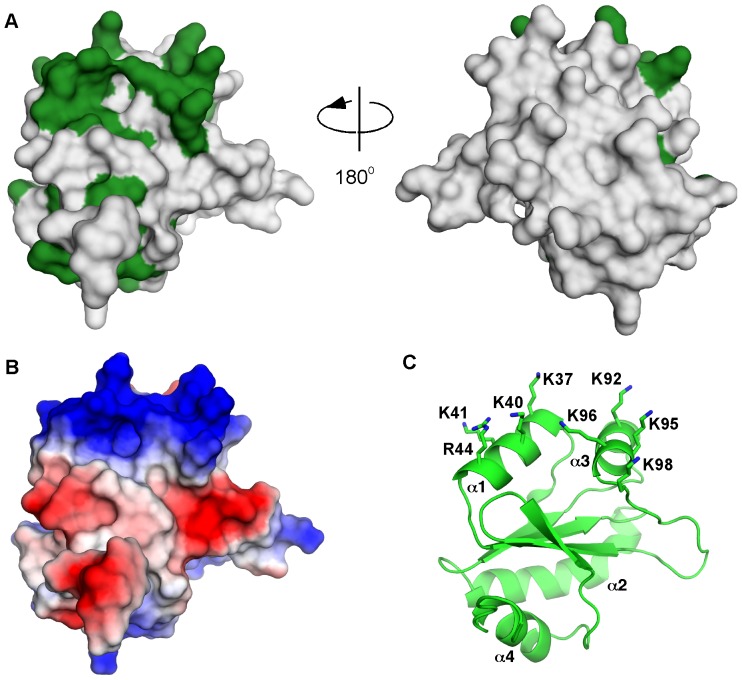
The non-catalytic domain of PDIR contains a conserved positively charged surface. (A) Mapping of sequence conservation on the surface of the human PDIR domain; invariant residues are colored green. (B) Surface charge; the positively charged (blue) surface coincides with the conserved region. Negative charge is in red. (C) The conserved lysine and arginine residues are located on helices α1 and α3. The domain orientation in panels (B) and (C) is identical to that in the left view of the panel (A).

We calculated the sequence conservation in the non-catalytic PDIR domain and mapped it onto the structure to identify the functionally important surfaces (Fig. 4A). The most conserved surface patches correspond to helices α1 and α3, which form a hydrophobic pocket to bind unfolded protein stretches in the non-catalytic domains of other PDIs [25]. The surface in PDIR is highly positively charged due to the presence of conserved lysine and arginine residues: Lys37, Lys40, Lys92, Lys95, Lys96 and Arg44 (Fig. 4BC). This pronounced charge strongly suggests that the surface does not bind hydrophobic polypeptides; rather, it is adapted for interactions with negatively charged proteins and may participate in PDIR substrate recruitment. Functional electrostatic interactions have been previously observed between ER proteins. The most notable example is the negatively charged P-domain of lectin chaperones calreticulin and calnexin, which bind to the positively charged surfaces of the protein disulfide isomerase ERp57 [26], [27] and the peptidyl-prolyl cis-trans isomerase CypB [11]. The presence of the conserved positively charged surface in the non-catalytic domain of PDIR led us to hypothesize that this domain is responsible for the PDIR binding to calreticulin, observed by mass spectrometry [8].

### Non-catalytic domain of PDIR interacts with the P-domain of calreticulin

**Figure 5:**
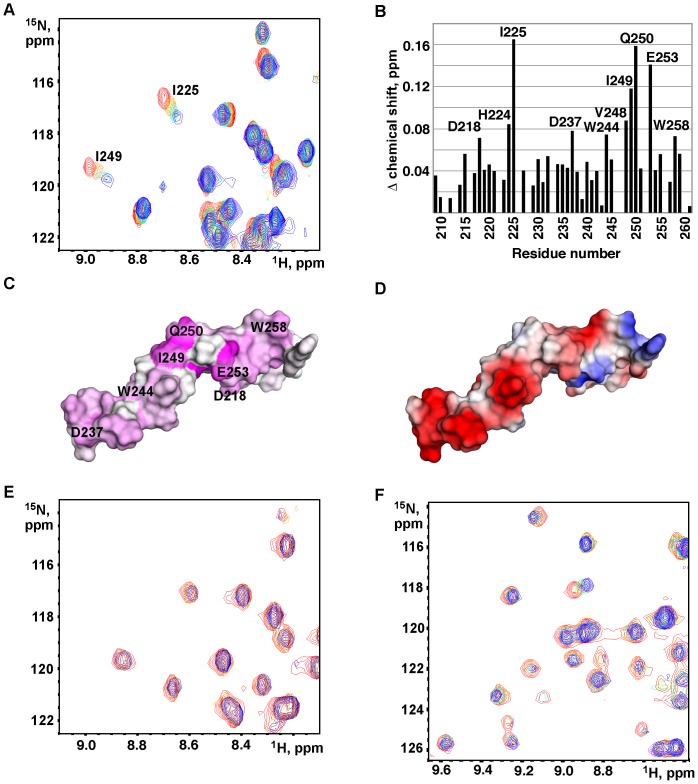
The PDIR non-catalytic domain binds to the P-domain of calreticulin. (A) Downfield region of HSQC spectra of ^15^N-labeled P-domain (residues 211–261) titrated with increasing amounts of the non-catalytic domain of PDIR. The spectra show specific chemical shift changes for residues Ile225 and Ile249. (B) Plot of weighted-average ^1^H and ^15^N chemical shift changes in the ^15^N-labeled calreticulin P-domain upon addition of the unlabeled PDIR domain. (C) Mapping of the chemical shifts measured onto the NMR structure of the calreticulin P-domain (PDB code 1k9c). Magenta indicates a large chemical shift change (>0.1 ppm); white indicates no change detected. Residues showing chemical shift changes above 0.07 ppm are labeled. (D) Surface charge representation of the P-domain. Negative charge is shown in red, positive charge is in blue. (E) Titration of the ^15^N-labeled P-domain with the PDIR non-catalytic domain in the presence of 0.5 M ammonium sulfate. The overlay corresponds to the P-domain/PDIR molar ratio of 1∶0 (red), 1∶1 (yellow) and 1∶2 (blue). (F) Titration of the ^15^N-labeled PDIR non-catalytic domain with increasing amounts of unlabeled P-domain results in shifts and disappearance of a number of peaks. Overlay shows spectra at the PDIR/P-domain molar ratio of 1∶0 (red), 1∶1 (yellow), 1∶2 (cyan), 1∶4 (purple) and 1∶8 (blue).

To characterize the interaction between PDIR and calreticulin, we ^15^N-labeled the P-domain fragment of calreticulin (residues 211–261) and monitored the changes in the NMR spectrum upon addition of the non-catalytic domain of PDIR. Addition of the PDIR domain caused a number of shifts in the ^1^H-^15^N correlation spectrum of the P-domain, indicating binding between the proteins (Fig. 5A). The largest chemical shift changes were in Ile225, Ile249, Gln250 and Glu253 (Fig. 5B). Mapping of the changes onto the structure of the calreticulin P-domain [28] reveals that the most affected region is not the tip of the domain, which is the site of ERp57 and CypB binding, but rather the hinge region of the construct (Fig. 5CD). Two of the most affected residues, I225 and I249, form a short β-sheet that separates the tip module and the adjacent module of the P-domain. The extended binding surface on the P-domain likely reflects the large size of the positively charged surface of the PDIR domain. In order to test our hypothesis that the interactions between these domains are mostly driven by electrostatics, we repeated the NMR titration in a buffer of higher ionic strength that contains 0.5 M ammonium sulfate. The titration yielded negligible changes in the P-domain spectrum (Fig. 5E) suggesting that the interaction was severely diminished.

We also performed a reverse NMR titration using ^15^N-labeled PDIR non-catalytic domain (residues 29–150) and unlabeled P-domain of calreticulin. The ^1^H-^15^N correlation spectrum of the PDIR domain showed excellent dispersion of signals characteristic of a well-folded domain (Fig. 5E). Addition of the P-domain caused specific shifts and disappearance of a number of signals, confirming binding between the proteins (Fig. 5F).

## Discussion

Although PDIR was first discovered more than 15 years ago, to our knowledge, this is the first structural characterization for any part of PDIR. The non-catalytic domain is of interest because of its enigmatic function and low sequence similarity to proteins of known structure. The structural similarity of the PDIR non-catalytic domain to the N-terminal domains of ERp57 and yeast PDI suggests that the proteins evolved in parallel from a common precursor with four catalytic domains. Perhaps, the PDIR N-terminal domain lost its catalytic activity while gaining the ability to bind substrates and other chaperones. More intriguing is the occurrence and function of PDIR N-terminal domain in RNA methyltransferases in parasitic nematodes. While the exact function in roundworms is unknown, SpoU methylase domains from other proteins bind to stem-loops of target RNA. The positively charged residues of PDIR are conserved in the methylase proteins and likely increase the affinity for binding RNA substrates.

Our structure shows that the PDIR non-catalytic domain adopts a typical thioredoxin-like fold but with several distinct features. One of these is the presence of an intramolecular disulfide bridge between Cys85 and Cys94. These cysteines are strictly conserved in PDIR which suggests a structural requirement for the disulfide. A similar disulfide bridge is implicated in the stability of the third catalytic domain of ERp46 [22]. Secondly, analysis of crystal contacts identified a putative substrate-binding site in the non-catalytic domain. The side chains of Leu143 and Trp144 insert into this pocket; their interactions with the adjacent protein molecule may mimic binding of hydrophobic stretches of misfolded polypeptides with the identified surface. Finally, the structure reveals a highly conserved, positively charged surface centered on helices α1 and α3 that is abundant in invariantly conserved lysine and arginine residues. The high degree of conservation strongly implies a functional importance of this feature.

NMR titrations show that the PDIR non-catalytic domain interacts with the P-domain of the ER lectin chaperone calreticulin. This result is consistent with the recent study that identified calreticulin as a binding partner of PDIR [8]. Furthermore, we confirmed one of these interactions using purified proteins and further delineated the binding by attributing it to particular fragments of both proteins. It should be noted that although NMR titrations displayed a fast-exchange binding mode correlated with relatively weak affinity, other domains in both proteins could potentially strengthen this interaction. Measurements of affinity by surface plasmon resonance using full-length proteins previously resulted in K_d_ of 16 µM [7]. Because of the pronounced negative charge of the calreticulin P-domain, it is very likely that the interaction involves the conserved positively charged surface on the PDIR domain. Notably, electrostatic interactions appear to be an emerging theme in ER protein-protein interactions. Examples include interactions of the negatively charged P-domain of calreticulin with conserved, positively charged surfaces of ERp57 [27] and CypB [11]. CypB employs the same positive surface to interact with the negatively charged N-terminal tail of another protein disulfide isomerase, ERp72 [8]. Given the reported interaction between ERp72 and PDIR [8], it is reasonable to speculate that positively charged surface of PDIR interacts with ERp72 in the same way.

In conclusion, our results demonstrate a role for the PDIR non-catalytic domain as the primary binding site for the major ER chaperone calreticulin and possibly other proteins and substrates. The occurrence of PDIR-like sequences in methyltransferases in some nematodes hints at a broader function for the PDIR domain or is at least an unusual example of gene transfer.
